# Sexual dimorphism in the effects of maternal adipose tissue growth hormone receptor deficiency on offspring metabolic health

**DOI:** 10.1186/s13293-024-00676-2

**Published:** 2024-12-02

**Authors:** Liyuan Ran, Xiaoshuang Wang, Rui Ma, Haoan Wang, Yingjie Wu, Zichao Yu

**Affiliations:** 1 Shandong Provincial Hospital, School of Laboratory Animal & Shandong Laboratory Animal Center, Central Hospital Affiliated to Shandong First Medical University, Shandong First Medical University, Jinan, Shandong 250021 China; 2 Institute of Genome Engineered Animal Models for Human Diseases, National Center of Genetically Engineered Animal Models for International Research, Dalian Medical University, Dalian, Liaoning 116044 China

**Keywords:** Growth hormone receptor, Adipose tissue, Sexual dimorphism, Metabolic homeostasis, Intergenerational effects, Adipose tissue developmental programming

## Abstract

**Background:**

The global incidence of obesity continues to rise, which increases the prevalence of metabolic diseases. We previously demonstrated the beneficial effect of adipose-specific growth hormone receptor (*Ghr*) knockout (KO) on metabolic parameters in male mice exposed to high fat diet. Although the effect of the growth hormone (GH) axis on lipid metabolism has been well studied, sexual dimorphism has not been considered. Furthermore, the effects of the GH axis on intergenerational adipose development are understudied. The present study aimed to evaluate whether adipose-specific *Ghr* knockout is associated with sex-specific differences in metabolic health of female offspring.

**Methods:**

*Ghr*^*flox/flox*^ (LL) mice were crossed with Adipoq-Cre mice to generate adipose-specific *Ghr* knockout (KO) mice. Physiological phenotype and fertility of female LL and KO mice were measured. Body weight, organ weight, glucose homeostasis, liver and adipose histology, hepatic triglycerides (TG) content, serum TG and low-density lipoprotein cholesterol (LDL-C) levels of female offspring were detected.

**Results:**

We found an increase in adipocyte size in female KO mice, but no change in glucose tolerance or insulin sensitivity. Adipose-specific *Ghr* deficiency impairs fertility in female KO mice. Maternal adipose-specific *Ghr* deficiency had a considerable beneficial effect on glucose metabolism in female offspring. The female offspring of the KO mice were protected against diet-induced obesity and the degree of hepatic steatosis and hyperlipidemia was reduced. The adipocyte size of the KO offspring did not change significantly despite the decrease in fat weight. Furthermore, the phenotypes of the offspring of LL mice fostered by the KO mothers differed from those of offspring remaining in the maternal nest.

**Conclusions:**

The findings of our study suggest that adipose GH axis plays a complex and important role in the intergenerational effects of metabolic health and adipocytes on offspring in a sex-specific manner. Future studies are needed to reveal the mechanisms of these sexually dimorphic phenotypes and the feasibility of providing new interventions for improving offspring metabolic health.

**Supplementary Information:**

The online version contains supplementary material available at 10.1186/s13293-024-00676-2.

## Abstract (plain language summary)

This study looks at how blocking a hormone called growth hormone (GH), which controls fat distribution and energy use, affects mothers and their children. We focused on female mice and blocked the action of GH in their fat tissue. Our results showed that when this hormone was blocked, the mothers gained more fat and had trouble with fertility, meaning it was harder for them to reproduce. This suggests that GH signaling pathway is important for both fat regulation and reproductive health.

Interestingly, blocking adipose GH signaling in the mothers had a protective effect on their female offspring. The daughters had better control over their blood glucose levels and were less likely to develop obesity or fatty liver problems, even when they ate a high-fat diet. This effect was mostly seen in the female offspring, not the males. We also found that the size of fat cells in the daughters did not increase, even though their fat mass was lower, suggesting that blocking GH changes how fat develops in the next generation.

In short, removing GH signaling from fat cells in mothers helps protect their daughters from weight gain and metabolic problems, while also showing how important GH is for reproduction. This research may lead to new ways to prevent obesity and health problems in children, especially in cases where the mother struggles with weight and fertility.

**Supplementary Information:**

The online version contains supplementary material available at 10.1186/s13293-024-00676-2.

## Highlights

**•** Adipose growth hormone receptor deficiency in female mice can increase adipose tissue mass and improve fasting blood glucose levels.
**•** Adipose growth hormone receptor deficiency impairs fertility in female mice.
**•** Maternal adipose growth hormone receptor disruption had a considerable beneficial effect to female offspring, including improved glucose homeostasis and resistance to diet-induced obesity and hepatic steatosis.
**•** The protective effects of maternal adipose growth hormone receptor deficiency were more pronounced in female offspring, suggesting sex-specific programming of metabolic health.
**•** Offspring of adipose growth hormone receptor deficiency mothers had lower fat mass without changes in fat cell size, indicating shifts in adipose tissue developmental programming.

**Supplementary Information:**

The online version contains supplementary material available at 10.1186/s13293-024-00676-2.

## Introduction

Obesity is a chronic disease, and the alarming increase in the global prevalence of obesity and related metabolic disorders has become a profound global health issue [1, 2]. The incidence of obesity is greater in women than in men and is partly heritable. The sex-specific differences in fat deposition patterns and lipid metabolism reflect the unique burdens women experience during gestation and lactation [3, 4]. The prevalence of childhood obesity has increased worldwide over recent decades [2]. Epidemiological and experimental studies have revealed a strong association between maternal obesity and the incidence of overweight offspring and metabolic syndrome in offspring [5–10]. Studies focused on the relationship between maternal obesity and offspring obesity, and its underlying mechanisms may thus provide new interventions for offspring health [11].

Growth hormone (GH), secreted from the anterior pituitary gland, plays a pivotal role in reproduction and in the regulation of development and growth in mammals both pre- and postnatal [12]. Previous evidence suggested that GH, or its downstream regulators, influences fetal development and that exposure to high levels of GH in utero significantly reduces offspring birth weights, possibly as a result of alterations in maternal metabolic pathways [13, 14]. The primary postnatal function of GH is to promote longitudinal growth, and GH is also involved in the regulation of lipid metabolism and homeostasis [15–17]. Adults with GH hyposecretion or a growth hormone receptor (GHR) gene mutation typically have an altered body composition and an accumulation of abdominal fat [18, 19]. We and others have studied the differential effects of GH on growth and tissue function using various knockout and transgenic mouse models that manipulate the disruption of *Ghr* in specific tissues, such as adipose tissue, to explore how GH can influence glucose homeostasis and lipid metabolism [20–22].

Adipose tissue, including both white and brown adipose tissue, is acquired mainly during the early developmental stages in mammals [23]. Adipose tissue is a key target of developmental programming in a sex- and adipose-depot-specific manner [24, 25]. It is well established that GH can regulate adipocyte differentiation and body fat accumulation [26]. However, its potential to affect intergenerational adipose development remains understudied. To date, the effect of adipose *Ghr* deficiency on lipid metabolism has been investigated only in males [22]. In addition, while the sexual dimorphism of GH-regulated gene expression in the liver has been reported [27, 28], sexual dimorphism in adipose tissue has not previously been considered. The present study aimed to characterize the effect of adipose-specific *Ghr* deficiency on female mice, with a focus on identifying potential sex-dependent differences in glucose and lipid metabolism and the influence of tissue-specific *Ghr* disruption on intergenerational effects.

## Materials and methods

### Animal models and diets

**Fig. 1:**
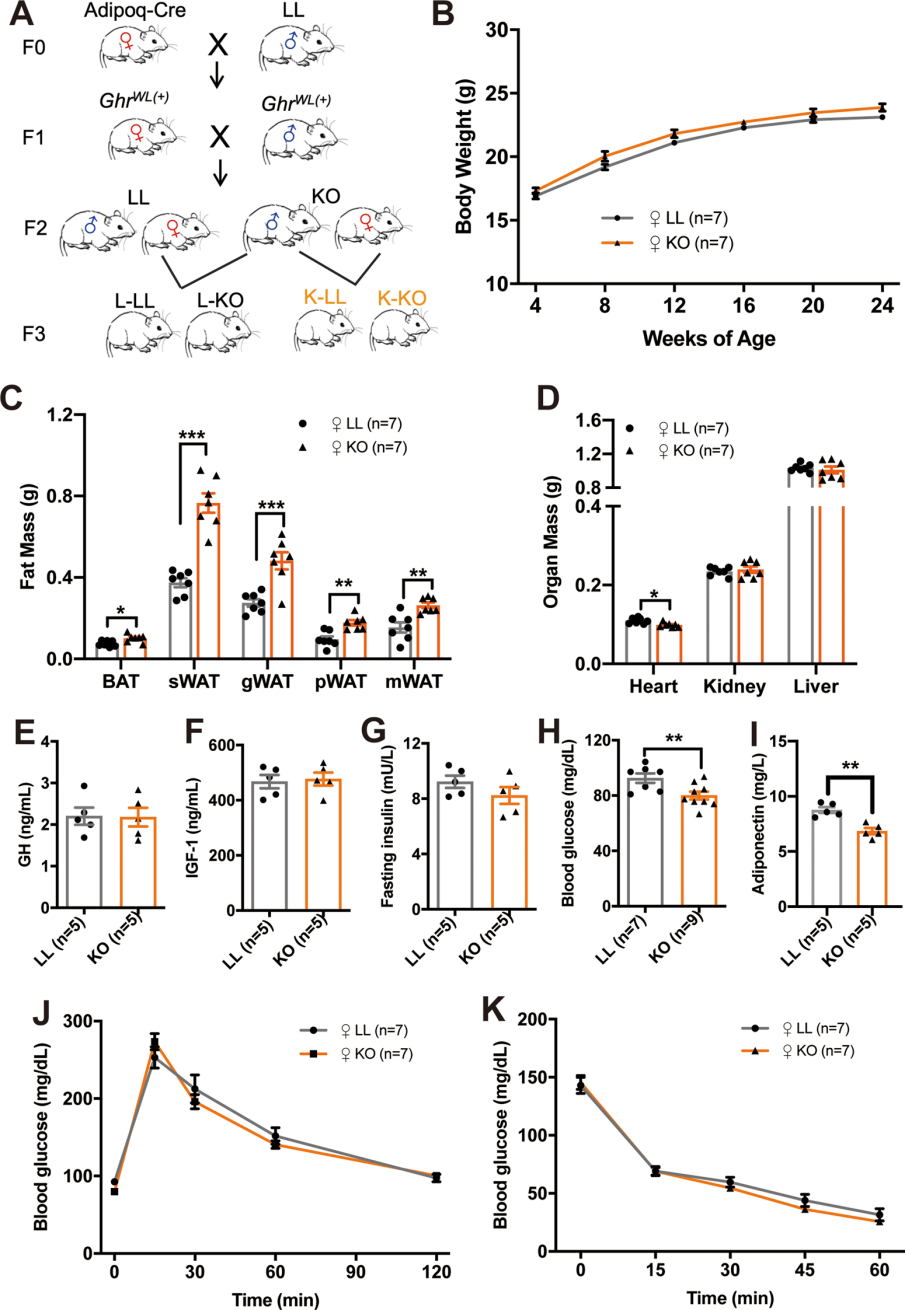
Characterization of female adipose-specific *Ghr* knockout mice. **A**: Schematic view of the mouse lines generation. **B**: Growth curves of female KO and LL mice after 24 weeks of RC feeding (*n* = 7 mice/group). **C**: Fat mass of 24-week-old female KO and LL mice (*n* = 7 mice/group). **D**: Organ mass of 24-week-old female KO and LL mice (*n* = 7 mice/group). **E-I**: Serum GH (**E**), IGF-1 (**F**), fasting insulin (**G**), blood glucose (**H**) and adiponectin (**I**) levels of 24-week-old female LL and KO mice (*n* = 5–9 mice/group). **J**: Glucose tolerance tests (GTTs) of 24-week-old female KO and LL mice (*n* = 7 mice/group). **K**: Insulin tolerance tests (ITTs) of 24-week-old female KO and LL mice (*n* = 7 mice/group)

All animal experiments were performed under the guidelines for the treatment of laboratory animals and were approved by the Committee on the Ethics of Animal Experiments of Dalian medical university. *Ghr*^*flox/flox*^ (LL) mice and adipose-specific *Ghr* knockout (KO) mice were used in this study. The LL mice which contain two LoxP sites flanking exon 4 of *Ghr* gene were previously generated by our group [29]. The KO mice were generated by crossing LL mice with Adipoq-Cre mice (Jackson Laboratory Stock No. 010803) (Fig. 1A, F0 to F2), and the LL littermates were used as the control (Table S1). To investigate the maternal effects of adipose tissue *Ghr* deletion on the metabolic health in adult female offspring, the male KO mice were respectively crossed with their female LL and KO littermates. The LL and KO offspring produced by female LL mice were defined as L-LL and L-KO, respectively; the LL and KO offspring produced by female KO mice were defined as K-LL and K-KO (Fig. 1A, F2 to F3, Table S2). A more detailed description of the mouse lines used in this study is provided in Supplementary Table S1 and Table S2 [30]. Mice were fed with regular chow (RC) (Jiangsu Xietong Medicine Bioengineering Co., Ltd., XTCON50J, Jiangsu, China) or high-fat diet (HFD) (Jiangsu Xietong Medicine Bioengineering Co., Ltd., XTHF60, Jiangsu, China). The composition of research diets was provided in Supplementary Table S3. All animals were maintained in a temperature- and humidity-controlled room on a 12-h light/dark cycle at a specific pathogen-free experimental animal center.

## Body weight, organ weight and serum analysis

Body weights were measured every two weeks during the entire experimental period. The mice were anesthetized using 2% avertin (0.1 mL/10 g body weight) (i.p.), and then blood and tissues were collected for further analysis. Organ were dissected and weighed on an analytical balance. The organs were then snap-frozen in liquid nitrogen before being stored at -80 °C or resuspended in formalin for histological analysis. The blood was collected in a 1.5 mL Eppendorf tube without additives or anti-coagulants and allowed to clot at room temperature, and the serum was separated from the whole blood by centrifugation at 4 °C at the speed of 3000 rpm for 15 min, the separated sera was stored at -80 °C until analysis is performed. The serum GH, IGF-1, insulin and adiponectin levels were measured in 5 mice from each group. ELISA kits for serum GH (www.lengton.com.cn, Cat# BPE20916), IGF-1 (www.lengton.com.cn, Cat# BPE20004), insulin (www.lengton.com.cn, Cat# BPE20687), follicle stimulating hormone (FSH, Jiangsu MeiMian, Cat# MM-23788), estradiol (E2, Jiangsu MeiMian, Cat# MM-1643) and luteinizing hormone (LH, Jiangsu MeiMian, Cat# MM-10404) were used to perform the serological analysis according to the manufacturer’s instructions. Total adiponectin levels were measured using an ELISA kit (Jiangsu MeiMian, Cat# MM-0547M1) according to the manufacturer’s instructions. The ELISA Kits (Jian Cheng Biological Engineering Institute, Nanjing, China) for alanine aminotransferase (ALT) (Cat# C009-1), aspartate aminotransferase (AST) (Cat# C010-1), triglyceride (TG) (Cat# A110-1) and low-density lipoprotein cholesterol (LDL-C) (Cat# A113-2) were used to perform the serological analysis according to the manufacturer’s instructions.

## Glucose tolerance test (GTT) and insulin tolerance test (ITT)

Blood glucose levels (*n* = 5 mice/group) were assessed following an overnight fast using a glucometer (Accu-Check Active, Roche). For the GTT (*n* = 5–8 mice/group), the mice were fasted overnight. Each mouse was weighed prior to intraperitoneal injection of glucose (2 mg/g body weight), after which blood glucose was detected at different time points. For the ITT (*n* = 5–8 mice/group), the mice were fasted for 4 h. Each mouse was weighed and intraperitoneally injected with 0.75 U/kg insulin, after which blood glucose was measured at different time points.

### Gene expression assessment by real-time qPCR

Total RNA was extracted from frozen tissues using an RNAiso Plus Kit (9109; Takara, Beijing, China) following the manufacturer’s protocol and then reverse transcribed using a PrimeScript™ RT reagent kit (RR047A, Takara). We quantified the expression levels of thermogenesis-related genes by real-time qPCR. The LL offspring of LL maternal parent with high-fat diet (L-LL HF) served as a control, and *Ucp1* and *Pgc1a* gene expression levels in the BAT of each group of mice were determined in reference to those levels in L-LL HF mice. The sequences of the primers used in this study are as follows: *Gapdh* forward: 5’-GGGCTGGCATTGCTCTCAATG-3’, reverse: 5’-CATGTAGGCCATGAGGTCCAC-3’; *Ucp1* forward: 5’-AAGACAGAAGAGCAT AGCATTCAC-3’, reverse: 5’-CCAGTCATACACTCCCACCTC-3’; *Pgc1a* forward: 5’-CCGAAGACACTACAGGTTCCATAG-3’, reverse: 5’-GGGAGGGAGAGAGGA GAGAGG-3’. *Gapdh* was used as the endogenous control. Amplification conditions of real-time qPCR were set according to the TransStart Tip Green qPCR SuperMix (AQ142, TransGen, Beijing, China): one cycle of 94 °C for 30s, followed by 40 cycles at 94 °C for 5 s and 60 °C for 30 s, then followed by dissociation stage. The real-time qPCR amplification and detection were run in the 7900HT Fast Real-Time PCR system (Applied Biosystems, Foster City, CA, USA). Eventually, the mRNA levels of selected genes were calculated after normalization to *Gapdh* by using the 2^-ΔΔCt^ algorithm.

### Histology and pathological analyses

Tissues were collected and fixed with 10% formalin for at least 24 h. Subsequently, the tissues were washed and stored in 70% ethanol until they were embedded in paraffin and sectioned at 8 μm intervals for hematoxylin-eosin (H&E) staining. For Oil red O staining, liver tissues were sucrose dehydrated and cut into 10 μm sections at -20^o^C, followed by staining with Oil red O (D027, Jian Cheng Biological Engineering Institute, Nanjing, China). The slides were observed using a Nikon Ni-E microscope and KF-PRO-005 MAGScanner to obtain images. The average area of adipocytes was analyzed using ImageJ software on H&E-stained slides from at least three 20× fields of three mice per genotype. Each of the 3 continuous sections of the mouse ovaries (5 mice of each genotype) was stained using hematoxylin-eosin, and the follicles were counted. The follicles were classified into distinct stages based on previously established standards [31].

### Liver TG assay

Frozen liver tissues (*n* = 4–5/group, 20 mg/mice) were homogenized in 0.18 ml ethanol in ice bath. The samples were centrifuged at 2500 rpm for 10 min, and the supernatants were collected to determine liver TG levels with a test kit (A110-1; Jian Cheng Biological Engineering Institute, Nanjing, China) according to the manufacturer’s instructions.

### Statistical analysis

All the data are shown as the means ± SEMs. GraphPad Prism (GraphPad Software, Inc., USA, version 8.4.0) was used for statistical calculations. Significance was determined by a two-tailed unpaired t test, Tukey’s multiple comparison test, ANOVA with the Tukey’s *post hoc* correction. The statistical significance threshold was set at *p* < 0.05. The statistical significance was indicated by *p < 0.05, **p < 0.01 and ***p < 0.001.

## Results

### Characterization of female adipose-specific *ghr* knockout mice

We previously created an adipose-specific *Ghr* gene knockout (KO) mouse model to study the effects of adipose-specific *Ghr* deficiency on the growth, metabolism and tissue function of male KO mice in the F2 generation [22]. Here, we focused on female KO mice in the F2 generation and female offspring in the F3 generation (Fig. 1A). The bodyweight of female KO mice in the F2 generation had no significant different with LL mice (Fig. 1B). Compared with those in the LL mice, the weights of different adipose depots in the female KO mice were greater (Fig. 1C) (*n* = 7 females, multiple t test; BAT: t = 2.889, *p* = 0.013; sWAT: t = 7.447, *p <* 0.001; gWAT: t = 4.539, *p <* 0.001; pWAT: t = 4.144, *p* = 0.0013; mWAT: t = 3.380, *p* = 0.0023), whereas the weight of the heart was lower (*n* = 7 females, multiple t test; Heart: t = 3.046, *p* = 0.01). No change in the weight of the kidney or liver was observed (Fig. 1D) (*n* = 7 females, multiple t test; Kidney: t = 0.5378, *p* = 0.6005; Liver: t = 0.5246, *p =* 0.6094).The mass of fat depots and other organs were corrected by body weight (Figure S1A and S1B) (Figure S1A: *n* = 7 females, multiple t test; BAT: t = 2.441, *p* = 0.031; sWAT: t = 6.616, *p <* 0.001; gWAT: t = 4.028, *p* = 0.0012; pWAT: t = 3.793, *p* = 0.0025; mWAT: t = 3.244, *p* = 0.007) (Figure S1B: *n* = 7 females, multiple t test; Heart: t = 3.239, *p* = 0.0071; Kidney: t = 0.6048, *p* = 0.5566; Liver: t = 1.320, *p =* 0.2116). Serological analysis indicated that adipose-specific *Ghr* deficiency did not affect the circulating levels of GH or IGF-1 or fasting insulin in female mice (Fig. 1E-G) (*n* = 5 females, two-tailed unpaired t test; GH: t = 0.08473, *p* = 0.9346; IGF-1: t = 0.2781, *p* = 0.7880; Insulin: t = 1.313, *p* = 0.2257), but female KO mice had lower fasting blood glucose (Fig. 1H) (*n* = 7–9 females, two-tailed unpaired t test; t = 3.389, *p* = 0.005) and adiponectin levels (Fig. 1I) (*n* = 5 females, two-tailed unpaired t test; t = 4.950, *p =* 0.001). Glucose tolerance tests (GTTs) and insulin tolerance tests (ITTs) revealed no difference between female KO mice and control littermates (Fig. 1J and K). Overall, these data indicated that *Ghr* deficiency in female mice has no effect on body weight. However, it can increase adipose tissue mass and improve fasting blood glucose levels.

All the mice were studied at 24 weeks of age unless otherwise stated. Adipoq-Cre: *Tg(Adipoq-cre)1Evdr* (JAX stock #010803); LL: *Ghr*^*flox/flox*^ mice; KO: adipose-specific *Ghr* knockout mice; L-LL: LL offspring of LL maternal parent; L-KO: KO offspring of LL maternal parent; K-LL: LL offspring of KO maternal parent; K-KO: KO offspring of KO maternal parent. BAT: brown adipose tissue; WAT: white adipose tissue; sWAT: subcutaneous WAT; gWAT: gonadal WAT; pWAT: perigonadal WAT; mWAT: mesenteric WAT. GH: growth hormone; IGF-1: insulin-like growth factor 1. All the values are presented as the means ± SEMs. The statistical significance was indicated by *p < 0.05, **p < 0.01 and ***p < 0.001.

### Adipose-specific *ghr* deficiency impairs fertility in female KO mice

**Fig. 2:**
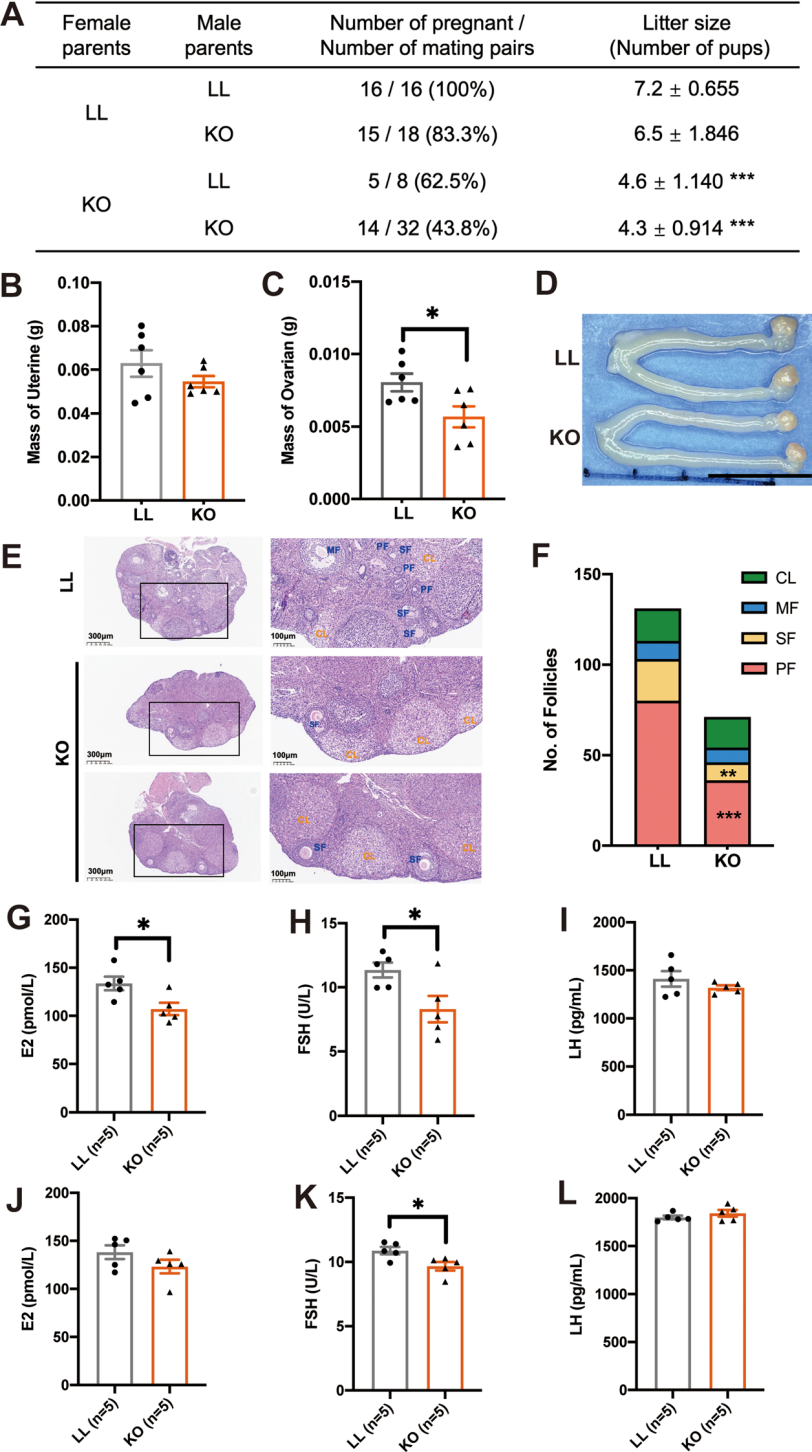
Adipose-specific *Ghr* deficiency impairs fertility in female KO mice. **A**: Evaluation of fertility in KO and LL mice. **B** and **C**: Uterine (**B**) and ovarian (**C**) weights of 10-week-old female KO and LL mice (*n* = 6 mice/group). **D**: Representative image of uterus and ovaries from 10-week-old female LL and KO mice. Scale bar: 1 cm. **E**: Representative photos of ovarian sections stained with H&E. **F**: Follicular quantification in each stage of 10-week-old female LL and KO mice. Follicles were counted on serial ovarian sections after H&E staining. **G-I**: Serum E2 (**G**), FSH (**H**) and LH (**I**) levels of 10-week-old female LL and KO mice (*n* = 5 mice/group). **J-L**: Serum E2 (**J**), FSH (**K**) and LH (**L**) levels of 16-week-old female LL and KO mice (*n* = 5 mice/group). PF: primary follicle; SF: secondary follicle; MF: mature follicle, and CL: corpus luteum. E2: estradiol; FSH: follicle stimulating hormone; LH: luteinizing hormone. All the values are presented as the means ± SEMs. The statistical significance was indicated by *p < 0.05, **p < 0.01 and ***p < 0.001.

Global GH deficiency could modify follicular development, ovarian maturation, ovulation rate, sexual maturation and pregnancy. To investigate the effects of adipose-specific *Ghr* deficiency on reproduction, we mated male LL mice and male KO mice with female LL or female KO mice respectively. The parental ages were 8–10 weeks. Our results showed that whether mated female KO mice with male LL or male KO mice, the reproductive function and the fertility rate were reduced significantly in female KO mice, and the number of pups per litter was lowest for the KO and KO mating pairs (Two-tailed unpaired t test; ♂LL x ♀LL vs. ♂LL x ♀KO: t = 6.453, *p <* 0.001; ♂KO x ♀LL vs. ♂KO x ♀KO: t = 3.984, *p <* 0.001). However, when the female LL mice mated with male LL or male KO mice, the litter size was unchanged (Two-tailed unpaired t test; t = 1.467, *p =* 0.153), which indicates that adipose-specific *Ghr* deficiency impairs fertility in female KO mice while the reproductive ability of male KO mice was not significantly affected (Fig. 2A). When the Adipo-Cre transgene is hemizygous in KO paternal mice, the LL maternal parent may give birth to L-LL and L-KO mice, while the KO maternal parent may give birth to K-LL and K-KO mice (Fig. 1A, F2 to F3). Therefore, we mated male KO mice with female LL or female KO mice to investigate the effects of different maternal genotypes on reproduction and offspring in the subsequent experiments. We assessed the reproductive organ weight and histomorphology of the ovaries of 10-week-old female mice. The weight of the uterus did not differ between female LL and KO mice (Fig. 2B) (*n* = 6 female mice, two-tailed unpaired t test; t = 1.259, *p =* 0.2366). However, compared with those in LL mice, the weight of the ovaries in female KO mice was significantly lower (Fig. 2C and D) (*n* = 6 female mice, two-tailed unpaired t test; t = 2.669, *p =* 0.0235). Follicular quantification showed a reduced percentage of primary follicles (PF, two-tailed unpaired t test; t = 6.016, *p <* 0.001) and secondary follicles (SF, two-tailed unpaired t test; t = 4.333, *p* = 0.002) in female KO mice while the number of mature follicles (MF, two-tailed unpaired t test; t = 0.5898, *p* = 0.5716) and corpus luteum (CL, two-tailed unpaired t test; t = 0.1741, *p* = 0.8661) has no significantly difference between LL and KO mice (Fig. 2E and F). These findings indicate that fertility defects in female KO mice are likely due to underdeveloped ovaries and impaired early follicular development. We measured the serum follicle stimulating hormone (FSH), estradiol (E2) and luteinizing hormone (LH) levels in 10-week-old and 16-week-old female mice separately to see whether sex hormone affect the fertility function of KO mice. We found that the E2 and FSH levels in 10-week-old female KO mice were significantly lower than those in LL mice (Fig. 2G and H) (*n* = 5 females, two-tailed unpaired t test; E2: t = 2.794, *p =* 0.0234; FSH: t = 2.579, *p =* 0.0327), while LH showed no significant changes (Fig. 2I) (*n* = 5 females, two-tailed unpaired t test; t = 1.093, *p =* 0.306). Female KO mice at 16 weeks of age still have lower FSH levels than LL mice (Fig. 2K) (*n* = 5 females, two-tailed unpaired t test; t = 2.800, *p =* 0.0232) but there is no significant difference in E2 and LH levels (Fig. 2J and L) (*n* = 5 females, two-tailed unpaired t test; E2: t = 1.484, *p =* 0.1762; LH: t = 1.118, *p =* 0.2959). These results are consistent with the fertility rate and histological staining, indicating that a decrease in E2 and FSH levels are probably a reason for the reduction of the reproductive function and the fertility rate in female KO mice, although the exact mechanism involved in the reduction of sex hormone remains unclear.

### Maternal adipose-specific *ghr* deficiency increases body fat mass and adipocyte size in female offspring

**Fig. 3:**
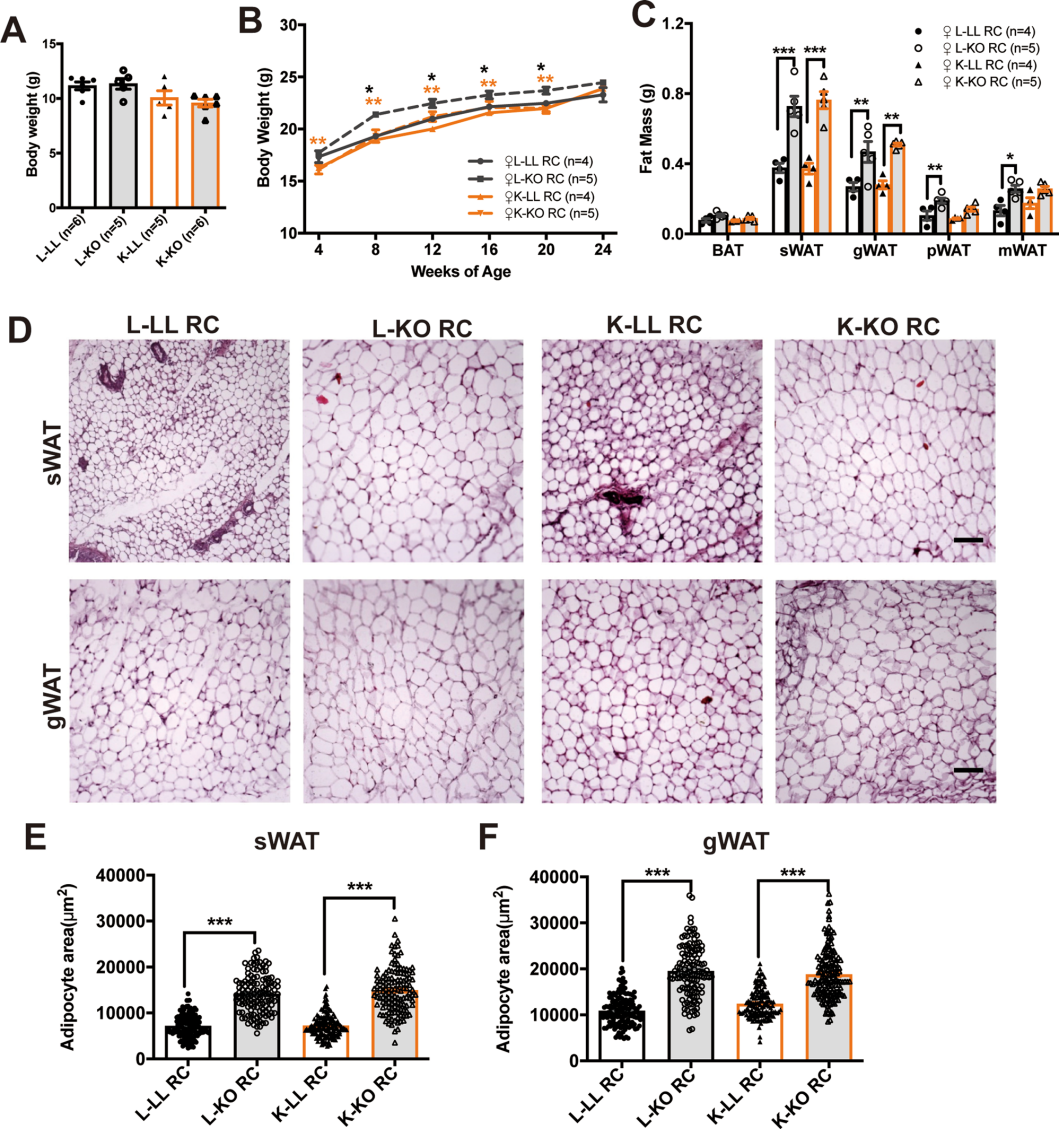
Maternal adipose-specific *Ghr* deficiency increases body fat mass and adipocyte size in female offspring fed regular chow. **A**: Body weights of 3-week-old female offspring of KO and LL maternal mice (*n* = 5–6 mice/group). **B**: Growth curves of female offspring of KO and LL maternal mice during RC feeding (*n* = 4–5 mice/group). **C**: Fat tissue weight of female offspring of KO and LL maternal mice under RC feeding (*n* = 4–5 mice/group). **D**: H&E staining of WAT sections. Scale bar: 100 μm. **E** and **F**: Quantification of the adipocyte area in sWAT (**F**) and gWAT (**G**). The average area of adipocytes was analyzed using ImageJ software on H&E-stained slides from at least three fields of three mice per genotype. All the values are presented as the means ± SEMs. The statistical significance was indicated by *p < 0.05, **p < 0.01 and ***p < 0.001.

GH plays a pivotal role in directing postnatal growth and regulating fat metabolism. We analyzed the body weight and fat mass of the offspring of KO and LL mothers. In the postnatal growth phase, the body weight of female K-KO mice (KO offspring of KO mothers) was 15.6% lower than that of female L-KO mice (KO offspring of LL mothers) at 3 weeks of age without significant difference (Fig. 3A) (One-way ANOVA; F (3, 18) = 3.550, *p =* 0.0354) (*post hoc* comparison, Tukey’s multiple comparisons test, ***p < 0.001). The body weight of the female L-KO mice was significantly greater than that of the female L-LL mice (LL offspring of LL mothers, black asterisk) and female offspring of the KO mothers (K-LL and K-KO, orange asterisk) during postnatal growth. Although there was no difference in body weight among four groups in 24-week old (Fig. 3B) (*n* = 4–5, two-way ANOVA; F (3, 84) = 23.63; *p* < 0.0001) (*Post hoc* comparison, Tukey’s multiple comparisons test, *p < 0.05 from week 8). However, we found that the maternal genotype did not affect bodyweight in male offspring (Figure S2A) (Figure S2A: *n* = 4–5, two-way ANOVA; F (3, 89) = 0.3895; *p =* 0.9786) (*Post hoc* comparison, Tukey’s multiple comparisons test). The white adipose tissue (WAT) of the KO mice was significantly heavier than that of the LL mice in the two groups of female offspring (Fig. 3C) (*n* = 4–5 females, one-way ANOVA for each fat depots; BAT: F (3, 14) = 3.353, *p =* 0.05; sWAT: F (3, 14) = 21.23, *p <* 0.0001; gWAT: F (3, 14) = 11.21, *p <* 0.0001; pWAT: F (3, 14) = 8.327, *p =* 0.002; mWAT: F (3, 14) = 6.911, *p =* 0.004) (*post hoc* comparison, Tukey’s multiple comparisons test, *p < 0.05, **p < 0.01 and ***p < 0.001). Fat index corrected by body weight showed the same result (Figure S1C) (*n* = 4–5 females, one-way ANOVA for each fat depots; BAT: F (3, 14) = 2.572, *p =* 0.095; sWAT: F (3, 14) = 17.57, *p <* 0.0001; gWAT: F (3, 14) = 11.38, *p* = 0.0005; pWAT: F (3, 14) = 6.553, *p =* 0.0054; mWAT: F (3, 14) = 6.495, *p =* 0.0056) (*post hoc* comparison, Tukey’s multiple comparisons test, *p < 0.05, **p < 0.01 and ***p < 0.001). Histology analysis revealed a significant increase in adipocyte size in L-KO and K-KO mice (Fig. 3D). The percentage of subcutaneous WAT (sWAT) adipocytes in L-KO and K-KO mice was 49.5% and 51.6% greater, respectively, than that in littermate LL offspring (Fig. 3E) (One-way ANOVA; F (3, 551) = 203.6, *p <* 0.0001) (*post hoc* comparison, Tukey’s multiple comparisons test, ***p < 0.001). However, the percentages of gonadal WAT (gWAT) were 44.2% and 34%, respectively (Fig. 3F) (One-way ANOVA; F (3, 558) = 134.3, *p <* 0.0001) (*post hoc* comparison, Tukey’s multiple comparisons test, ***p < 0.001). Overall, these results indicated that a lack of *Ghr* in adipose tissue may increase fat mass and adipocyte size in mice fed regular chow, consistent with our previous study in male mice [22].

### The female offspring of KO mothers are resistant to dietary obesity, which improves glucose homeostasis

**Fig. 4:**
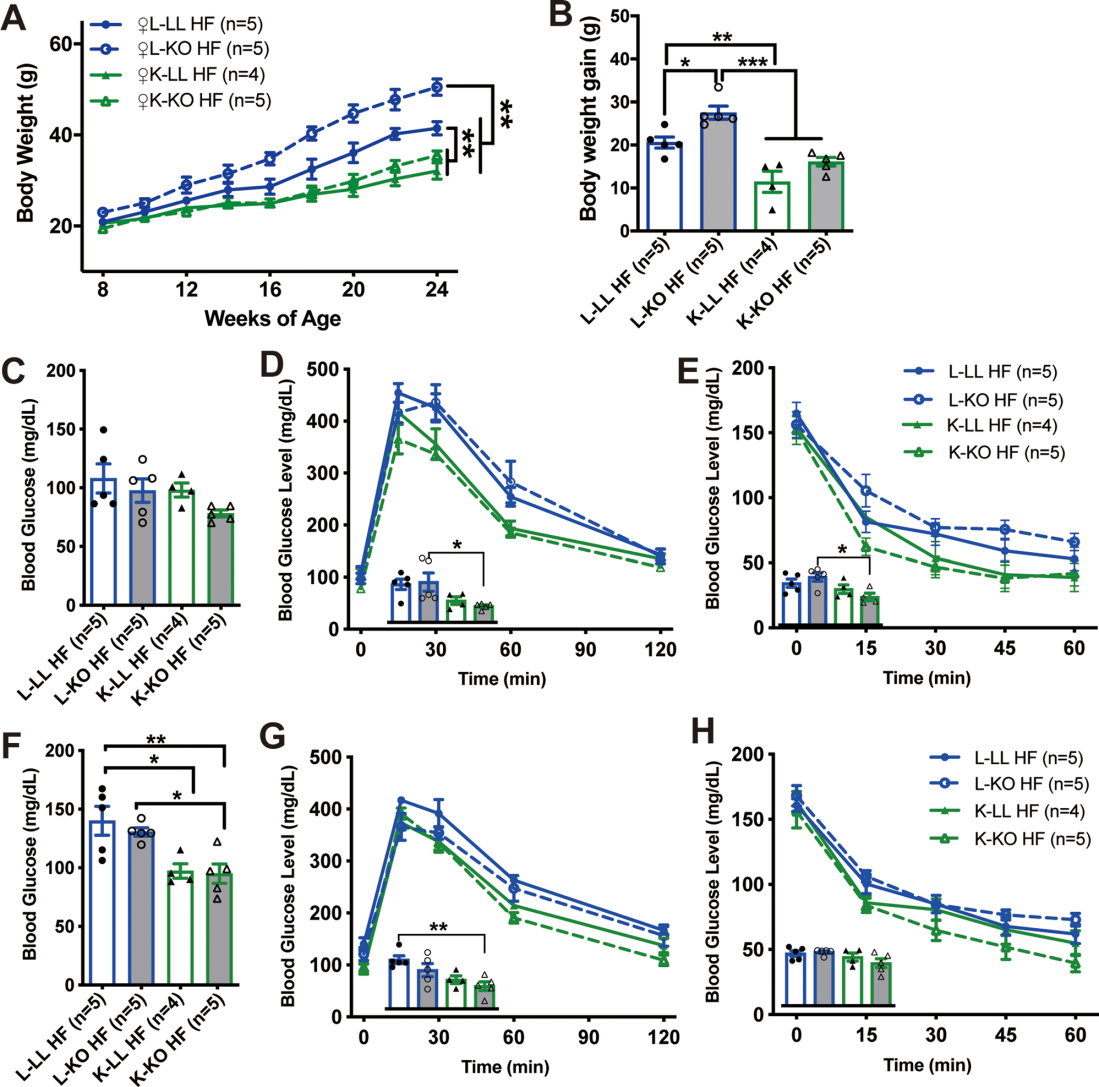
Female offspring of KO mothers are resistant to diet-induced obesity and glucose homeostasis is improved. **A**: Growth curves of female offspring of KO and LL maternal mice during HF feeding (*n* = 4–5 mice/group). **B**: Increase in body weight (24-week vs. 8-week) of female offspring of KO and LL maternal mice (*n* = 4–5 mice/group). **C**: Fasting blood glucose levels of female offspring mice fed a high-fat diet for 8 weeks (*n* = 4–5 mice/group). **D** and **E**: GTTs (**D**) and ITTs (**E**) results for female offspring mice fed a high-fat diet for 8 weeks (*n* = 4–5 mice/group). Inset: The area under the curve. **F**: Fasting blood glucose levels of female offspring mice fed a high-fat diet for 16 weeks (*n* = 4–5 mice/group). **G** and **H**: GTTs (**G**) and ITTs (**H**) results for female offspring mice fed a high-fat diet for 16 weeks (*n* = 4–5 mice/group). Inset: The area under the curve. All the values are presented as the means ± SEMs. The statistical significance was indicated by *p < 0.05, **p < 0.01 and ***p < 0.001.

In our previous study [22], we found that the absence of *Ghr* in fat exacerbated diet-induced obesity in male KO mice, and the maternal genotype did not affect this outcome in male offspring (Figure S2B and S2C) (Figure S2B: *n* = 6–8, two-way ANOVA; F (3, 125) = 12.43, *p <* 0.0001) (*Post hoc* comparison, Tukey’s multiple comparisons test, *p < 0.05 from week 20) (Figure S2C: One-way ANOVA; F (3, 24) = 9.377, *p =* 0.0003) (*post hoc* comparison, Tukey’s multiple comparisons test, *p < 0.05 and **p < 0.01). Consistent with this, when the mothers were LL mice, the body weights of the L-LL and L-KO female mice were significantly greater after with the mice were fed a HF diet, while the L-KO female mice were more obese (Fig. 4A and B). In contrast, when the matrilineal parent was a KO mouse, both K-LL and K-KO female mice were resistant to dietary obesity (Fig. 4A and B) (Fig. 4A: *n* = 4–5, two-way ANOVA; F(3, 135) = 106.1; *p* < 0.0001) (*Post hoc* comparison, Tukey’s multiple comparisons test, *p < 0.05 from week 12) (Fig. 4B: *n* = 4–5, one-way ANOVA; F (3, 15) = 18.89, *p <* 0.0001) (*post hoc* comparison, Tukey’s multiple comparisons test, *p < 0.05, **p < 0.01 and ***p < 0.001). After feeding a HF diet for 8 weeks, the fasting blood glucose levels have no significantly different in four groups (K-KO, 78.12 ± 6.44 mg/dL; K-LL, 98.1 ± 11.89 mg/dL; L-LL, 108 ± 27.9 mg/dL; L-KO, 97.56 ± 22.24 mg/dL) (Fig. 4C) (*n* = 4–5, one-way ANOVA; F (3, 15) = 2.057, *p =* 0.1491). GTTs and ITTs revealed that female K-KO mice were more responsive to glucose challenge (Fig. 4D) (*n* = 4–5, one-way ANOVA; F (3, 15) = 4.237, *p* = 0.0234) (*post hoc* comparison, Tukey’s multiple comparisons test, *p < 0.05) and more sensitive to insulin stimulation than L-KO mice (Fig. 4E) (*n* = 4–5, one-way ANOVA; F (3, 14) = 4.154, *p =* 0.0267) (*post hoc* comparison, Tukey’s multiple comparisons test, *p < 0.05). After prolonged HF diet induction for 16 weeks, the fasting blood glucose levels of K-LL and K-KO female mice were lower than those of L-LL and L-KO female mice (K-LL, 91.44 ± 16.76 mg/dL; K-KO, 95.04 ± 18.51 mg/dL; L-LL, 140.04 ± 27.58 mg/dL; L-KO, 133.2 ± 6.06 mg/dL) (Fig. 4F) (*n* = 4–5, one-way ANOVA; F (3, 15) = 7.361, *p* = 0.0029) (*post hoc* comparison, Tukey’s multiple comparisons test, *p < 0.05 and **p < 0.01). In addition, K-KO female mice responded better to the glucose challenge (Fig. 4G) (*n* = 4–5, one-way ANOVA; F (3, 15) = 5.742, *p* = 0.008) (*post hoc* comparison, Tukey’s multiple comparisons test, **p < 0.01) despite not exhibiting an advantage in insulin sensitivity (Fig. 4H) (*n* = 4–5, one-way ANOVA; F (3, 15) = 2.322, *p =* 0.1165). However, we found that the maternal genotype did not affect the fasting blood glucose levels in male offspring with HF diet (Figure S2D and S2E) (Figure S2D: *n* = 6 males, One-way ANOVA; F (3, 20) = 17.51, *p* < 0.0001) (*post hoc* comparison, Tukey’s multiple comparisons test, *p < 0.05, **p < 0.01 and ***p < 0.001) (Figure S2E: *n* = 6 males, One-way ANOVA; F (3, 20) = 7.810, *p =* 0.0012) (*post hoc* comparison, Tukey’s multiple comparisons test, *p < 0.05 and **p < 0.01). These data suggest that maternal adipose *Ghr* disruption has a beneficial effect only on body weight and glucose metabolism in female offspring fed a HF diet.

### Maternal adipose-specific *ghr* deficiency inhibits adipose hyperplasia and alleviates hepatic steatosis and hyperlipidemia in female offspring

**Fig. 5:**
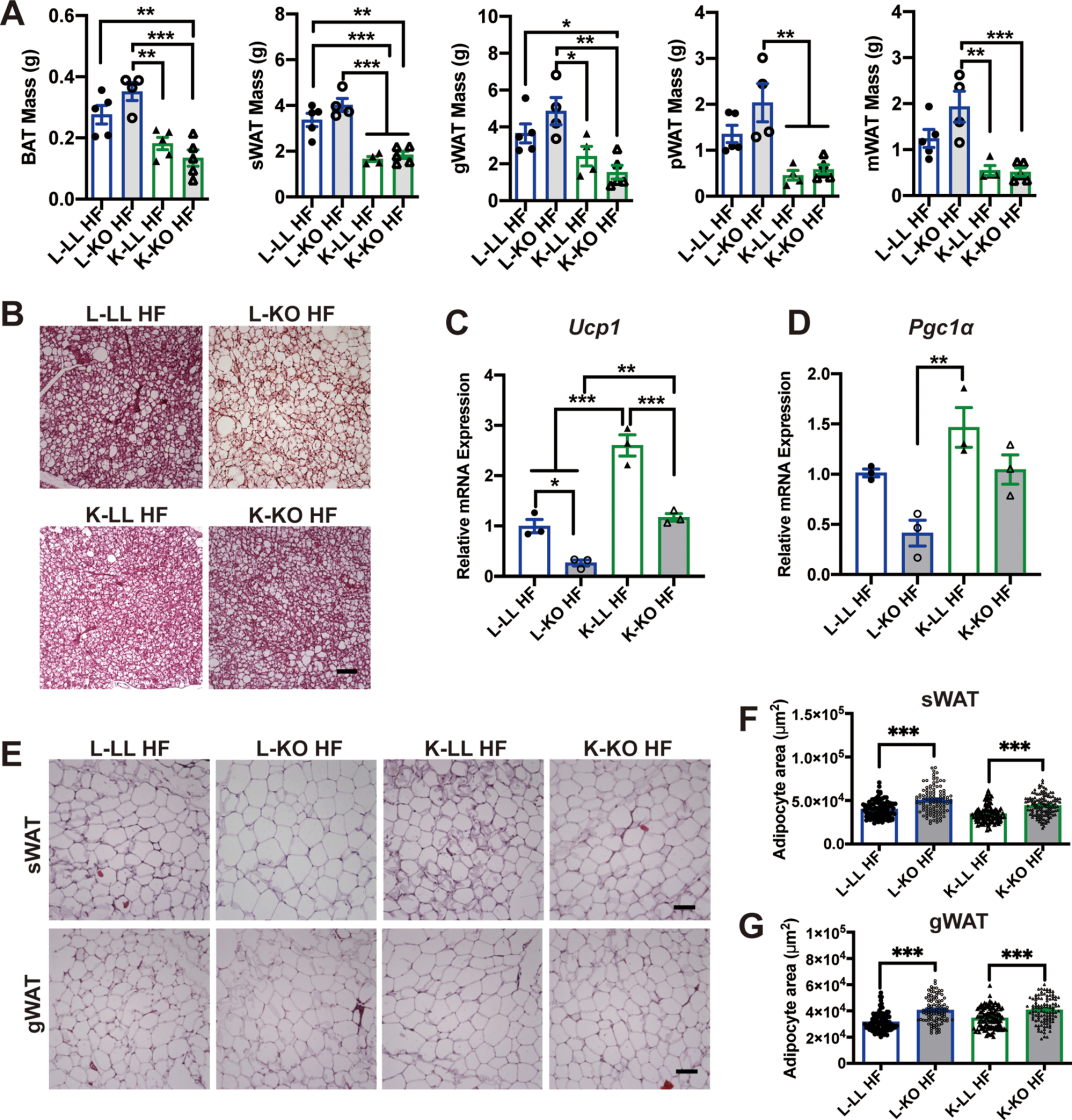
Maternal adipose-specific *Ghr* deficiency inhibits adipose hyperplasia during high-fat diet feeding. **A**: Fat tissue weight of female offspring of KO and LL maternal mice after high-fat diet feeding for 16 weeks (*n* = 4–5 mice/group). **B**: H&E staining of BAT sections. Scale bar: 100 μm. **C**s**D**: Relative mRNA expression levels of *Ucp1* (**C**) and *Pgc1a* (**D**) in BAT (*n* = 3 mice/group). **E**: H&E staining of sWAT and gWAT sections. Scale bar: 100 μm. **F** and **G**: Quantification of the adipocyte area in sWAT (**F**) and gWAT (**G**). *Ucp1*: uncoupling protein 1; *Pgc1α*: peroxisome proliferator-activated receptor gamma coactivator 1 alpha. The average area of adipocytes was analyzed using ImageJ software on H&E-stained slides from at least three fields of three mice per genotype. All the values are presented as the means ± SEMs. The statistical significance was indicated by *p < 0.05, **p < 0.01 and ***p < 0.001.

To evaluate the effect of maternal genotype on adipose tissue parameters, we compared fat mass among four different offspring. Consistent with the body weight, fat mass across various locations was significantly greater in the L-LL and L-KO female mice after HF induction, while fat mass was less increased in the K-LL and K-KO female mice (Fig. 5A) (*n* = 4–5 females, one-way ANOVA for each fat depots; BAT: F (3, 15) = 12.44, *p =* 0.0002; sWAT: F (3, 14) = 24.46, *p <* 0.0001; gWAT: F (3, 14) = 7.252, *p =* 0.0036; pWAT: F (3, 14) = 10.23, *p =* 0.0008; mWAT: F (3, 13) = 6.908, *p =* 0.0051) (*post hoc* comparison, Tukey’s multiple comparisons test, *p < 0.05, **p < 0.01 and ***p < 0.001). Because the body weights of the L-LL and L-KO female mice were significantly greater than K-LL and K-KO female mice under HF diet (Fig. 4A). We corrected the weight of fat depots with bodyweight. As shown in figure S1D, the fat index of K-LL and K-KO mice were still significantly lower than L-LL and L-KO female mice (*n* = 4–5 females, one-way ANOVA for each fat depots; BAT: F (3, 14) = 4.209, *p =* 0.0256; sWAT: F (3, 14) = 13.29, *p =* 0.0002; gWAT: F (3, 14) = 4.325, *p =* 0.0235; pWAT: F (3, 14) = 7.03, *p =* 0.0041; mWAT: F (3, 14) = 11.04, *p =* 0.0006) (*post hoc* comparison, Tukey’s multiple comparisons test, *p < 0.05, **p < 0.01 and ***p < 0.001). We previously showed that adipose-specific *Ghr* deficiency leads to lipid accumulation in brown adipose tissue (BAT) and impaired cold tolerance [22]. Here, we found lipid droplet expansion and accumulation in the BAT of female L-LL and L-KO mice; however, the degree of whitening in female K-LL and K-KO mice was markedly reduced (Fig. 5B). In addition, the expression levels of thermogenic genes, such as *Ucp1* and *Pgc1α*, were decreased in the BAT of female L-KO HF mice. Compared to that in the offspring from the LL mothers, the expression of thermogenic genes was upregulated in female K-LL and K-KO mice (Fig. 5C and D) (One-way ANOVA; *Ucp1*: F (3, 8) = 53.53, *p <* 0.0001; *Pgc1α*: F (3, 8) = 9.608, *p* = 0.005) (*post hoc* comparison, Tukey’s multiple comparisons test, *p < 0.05, **p < 0.01 and ***p < 0.001). Obesity in both *Ghr* total knockout and adipose-specific *Ghr* knockout mice is attributed to fat expansion in terms of both the number and size of adipocytes [22, 32–35]. Therefore, we examined the histopathological features of sWAT and gWAT in these mice. As expected, the adipocytes of female L-KO and K-KO mice were significantly larger than those of female L-LL and K-LL mice fed a HF diet (Fig. 5E). Although fat mass was decreased in K-LL and K-KO mice, surprisingly, no significant difference in adipocyte size was observed between the offspring of the LL and KO matrilineal parents (Fig. 5E and G) (One-way ANOVA; sWAT: F (3, 420) = 33.29, *p <* 0.0001; gWAT: F (3, 401) = 27.66, *p <* 0.0001) (*post hoc* comparison, Tukey’s multiple comparisons test, ***p < 0.001). Thus, the reduction in adipose tissue mass in female K-LL and K-KO mice resulted from a decrease in the number of adipocytes rather than the size of adipocytes.

**Fig. 6:**
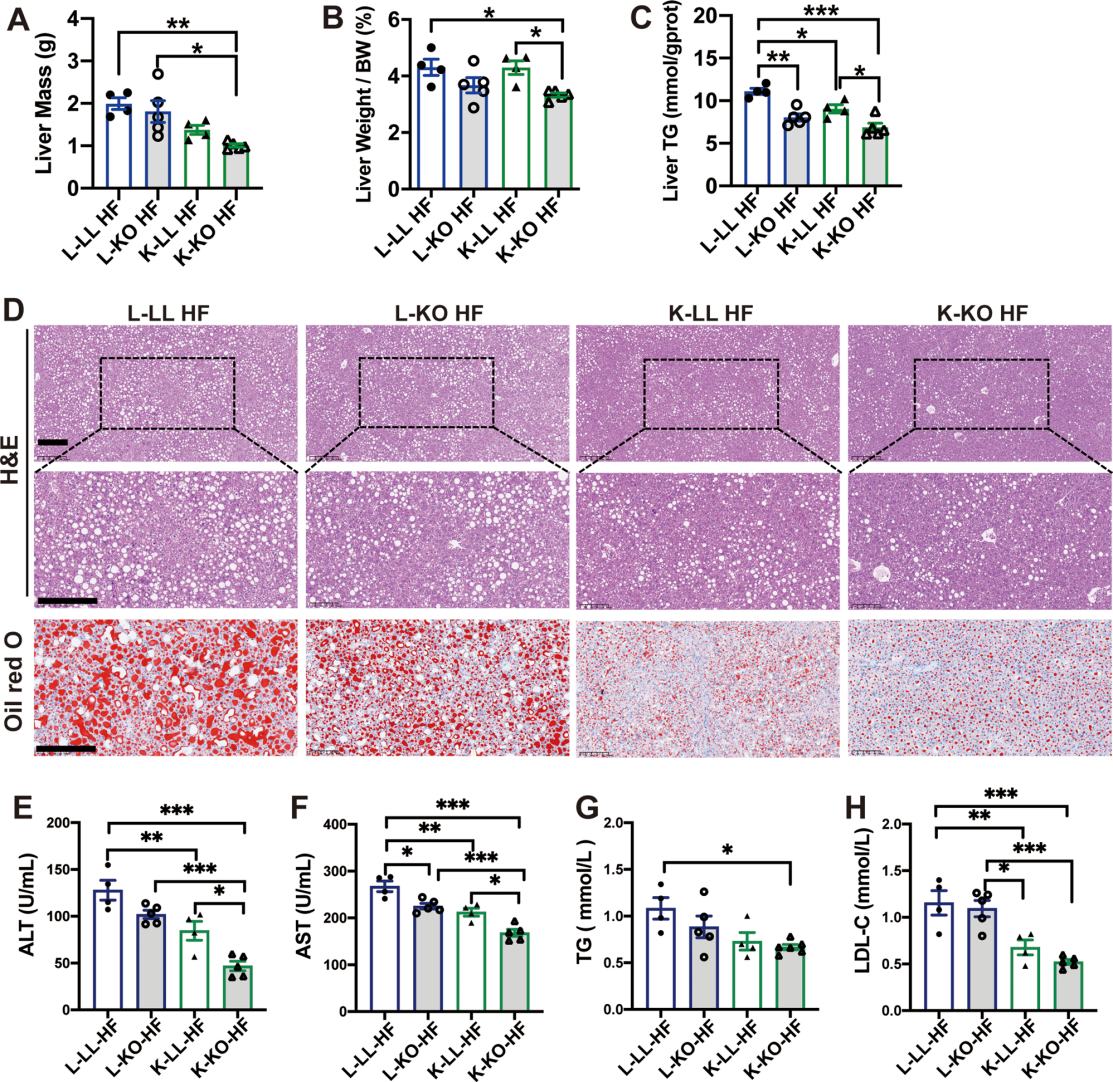
Maternal adipose-specific *Ghr* deficiency alleviates hepatic steatosis and hyperlipidemia in female offspring fed a high-fat diet. **A**: Liver mass of female offspring mice fed a high-fat diet for 16 weeks (*n* = 4–5 mice/group). **B**: Liver indices (liver weight/body weight%) of female offspring mice fed a HF diet for 16 weeks (*n* = 4–5 mice/group). **C**: Liver TG content of female offspring mice fed a HF diet for 16 weeks (*n* = 4–5 mice/group). **D**: H&E and Oil red O staining of liver sections from female offspring mice fed a RC or HF diet for 16 weeks. Bar: 200 μm. **E-H**: Serum ALT (**E**), AST (**F**), TG (**G**) and LDL-C (**H**) levels of female offspring mice fed a HF diet for 16 weeks (*n* = 4–5 mice/group). BW: body weight; TG: triglyceride; ALT: alanine aminotransferase; AST: aspartate aminotransferase; LDL-C: low-density lipoprotein cholesterol. All the values are presented as the means ± SEMs. The statistical significance was indicated by *p < 0.05, **p < 0.01 and ***p < 0.001.

As a major site for lipogenesis and lipid oxidation, the liver is the central engine modulating whole-body metabolic homeostasis and is vulnerable to nutritional damage. Organ weight analysis revealed reduced weights of the liver in female K-KO mice than L-LL and L-KO mice fed a HF diet (Fig. 6A) (*n* = 4–5 females, one-way ANOVA F (3, 14) = 7.310, *p =* 0.0035) (*post hoc* comparison, Tukey’s multiple comparisons test, *p < 0.05 and **p < 0.01). And the liver index of female K-KO mice was also significantly lower than those of L-LL HF and K-LL HF groups (Fig. 6B) (One-way ANOVA; F (3, 14) = 4.544, *p =* 0.0201) (*post hoc* comparison, Tukey’s multiple comparisons test, *p < 0.05 and **p < 0.01). Furthermore, compared to L-LL HF group, the liver triglyceride (TG) contents were brought down significantly by 27.5% in L-KO HF, 11.7% in K-LL HF and 38.1% in K-KO HF (Fig. 6C) (One-way ANOVA; F (3, 14) = 15.81, *p <* 0.0001) (*post hoc* comparison, Tukey’s multiple comparisons test, *p < 0.05, **p < 0.01 and ***p < 0.001). Histological staining revealed features of hepatic steatosis in the HF diet-fed group. A significant increase of hepatic steatosis marked by visible lipid droplets and ballooning hepatocytes was observed in both groups fed with HFD (Fig. 6D). More visible lipid droplets were observed in female L-LL and L-KO mice. Hepatic lipid accumulation was reduced in the K-LL and K-KO mice, especially in female K-KO mice (Fig. 6D). In the oil red O staining, the lipid droplets are colored into red. Liver tissues of L-LL HF mice and L-KO HF mice both showed abundant red area, further indicating massive lipids accumulation in the liver (Fig. 6D). The release of aspartate aminotransferase (AST) and alanine aminotransferase (ALT) frequently adapted as reflection of hepatocellular integrity. We found a significant reduction in relative activity of both AST and ALT of K-KO mice (Fig. 6E and F) (One-way ANOVA; AST: F (3, 14) = 24.25, *p <* 0.0001; ALT: F (3, 14) = 21.01, *p <* 0.0001) (*post hoc* comparison, Tukey’s multiple comparisons test, *p < 0.05, **p < 0.01 and ***p < 0.001). The influence of maternal adipose-specific *Ghr* deficiency on lipid profile of female offspring was examined by measuring the serum TG and low-density lipoprotein cholesterol (LDL-C) levels under HF diet condition. The results showed that the serum TG of K-KO HF mice was significantly lower than in L-LL HF mice (Fig. 6G) (One-way ANOVA; F (3, 14) = 4.167, *p =* 0.0247) (*post hoc* comparison, Tukey’s multiple comparisons test, *p < 0.05). Similarly, the serum LDL-C levels in K-LL and K-KO mice were also significantly lower than in female offspring of LL mice under HF diet condition (Fig. 6H) (One-way ANOVA; F (3, 14) = 13.78, *p =* 0.0002) (*post hoc* comparison, Tukey’s multiple comparisons test, *p < 0.05, **p < 0.01 and ***p < 0.001), confirming a suppressed development of hyperlipidemia in the offspring of KO mice.

### Swapping of the matrilineal parent during lactation reverses the phenotype of the female offspring

**Fig. 7:**
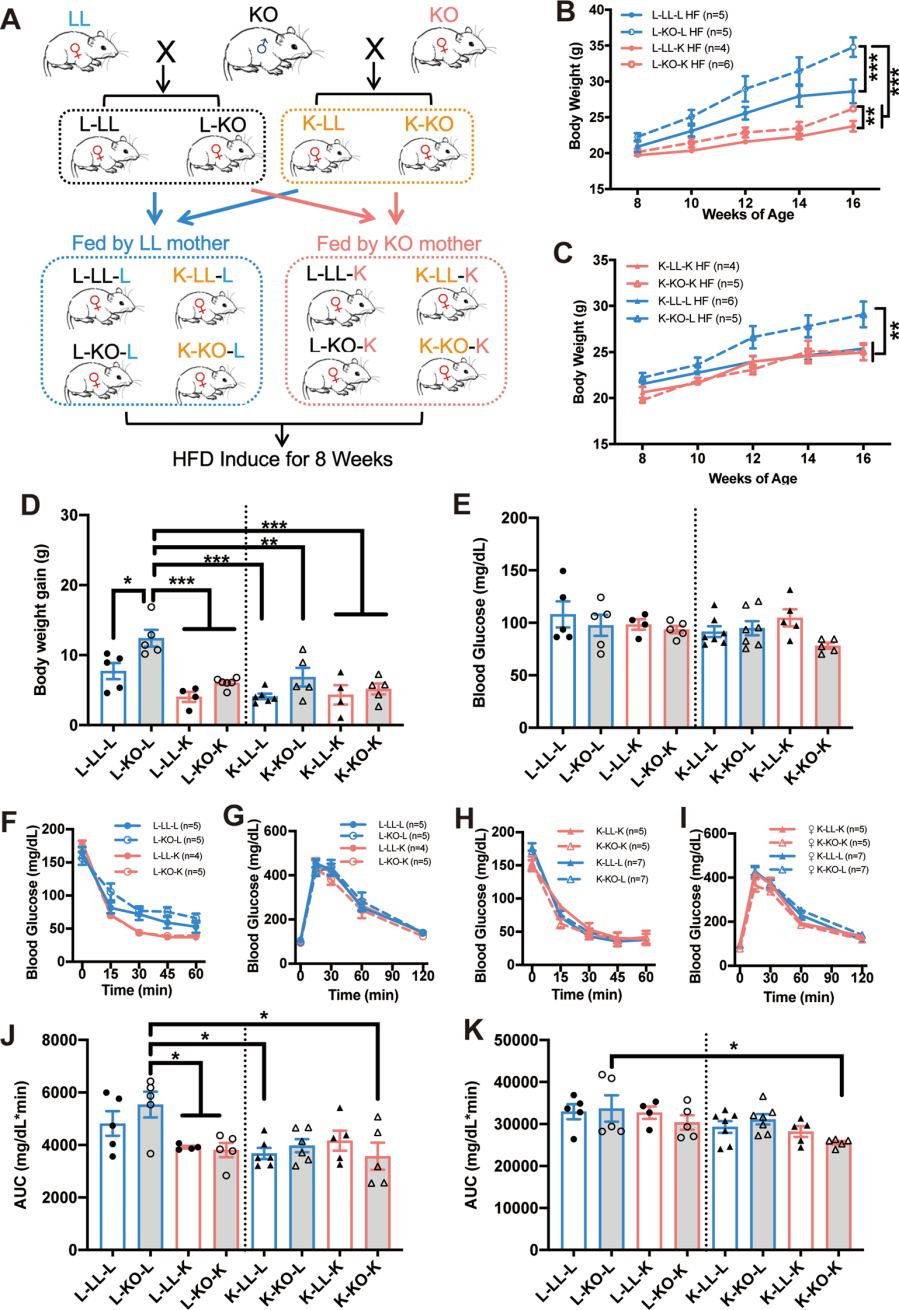
Swapping of the matrilineal parent during lactation reverses the phenotype of the female offspring. **A**: Schematic diagram of KO and LL mouse breeding and swapping of maternal feeding. **B**: Growth curves of female offspring of LL mice after switching maternal feeding (*n* = 4–6 mice/group). **C**: Growth curves of female offspring of KO mice after switching maternal feeding (*n* = 4–6 mice/group). **D**: Increase in the body weight of female offspring after switching the matrilineal parent (*n* = 4–7 mice/group). **E**: Blood glucose levels of female offspring after switching the matrilineal parent (*n* = 4–7 mice/group). **F** and **G**: ITTs (**F**) and GTTs (**G**) results for female offspring of LL maternal mice after swapping maternal feeding (*n* = 4–5 mice/group). s **I**: ITTs (**H**) and GTTs (**I**) results for female offspring of KO maternal mice after swapping maternal feeding (*n* = 5–7 mice/group). **J** and **K**: The area under the curves (AUCs) of the ITT (**J**) and GTT (**K**) (*n* = 4–7 mice/group). L-LL-L: LL offspring of LL maternal parent fed by LL mother; L-KO-L: KO offspring of LL maternal parent fed by LL mother; K-LL-L: LL offspring of KO maternal parent fed by LL mother; K-KO-L: KO offspring of KO maternal parent fed by LL mother; L-LL-K: LL offspring of LL maternal parent fed by KO mother; L-KO-K: KO offspring of LL maternal parent fed by KO mother; K-LL-K: LL offspring of KO maternal parent fed by KO mother; K-KO-K: KO offspring of KO maternal parent fed by KO mother. All the values are presented as the means ± SEMs. The statistical significance was indicated by *p < 0.05, **p < 0.01 and ***p < 0.001.

To verify that the body weight and metabolic health of the offspring are influenced by feeding from different matrilineal parents, we exchanged the mother of the first female filial generation in the early postnatal period. Weanling mice were acclimated for one week and then fed a HF diet until they were 16 weeks old (Fig. 7A). Surprisingly, when the female offspring of LL mice (L-LL and L-KO) were fed by a KO mother (L-LL-K and L-KO-K), they were also resistant to diet-induced obesity (Fig. 7B red line and Fig. 7D) (Fig. 7B: *n* = 4–6, two-way ANOVA; F(3, 79) = 43.02; *p* < 0.0001) (*Post hoc* comparison, Tukey’s multiple comparisons test, *p < 0.05 from week 10) (Fig. 7D: *n* = 4–6, one-way ANOVA; F (7, 32) = 8.907, *p <* 0.0001) (*post hoc* comparison, Tukey’s multiple comparisons test, *p < 0.05, **p < 0.01 and ***p < 0.001). K-LL-L mice (female LL offspring of KO mice fed by a LL mother) remained resistant to dietary obesity even when fed by a LL mother, but the body weight of K-KO-L mice (female KO offspring of KO mice fed by a LL mother) increased significantly (Fig. 7C) (Fig. 7C: *n* = 4–6, two-way ANOVA; F(3, 78) = 15.58; *p* < 0.0001) (*Post hoc* comparison, *n* = 4–5, Tukey’s multiple comparisons test, *p < 0.05 from week 12). Furthermore, the offspring of KO mothers exhibited reduced body weight gain (Fig. 7D, right side) (Fig. 7D: *n* = 4–6, one-way ANOVA; F (7, 32) = 8.907, *p <* 0.0001) (*post hoc* comparison, Tukey’s multiple comparisons test, *p < 0.05, **p < 0.01 and ***p < 0.001). To further investigate whether swapping feeding sources could influence glucose metabolism in female offspring, fasting blood glucose levels were measured. Blood glucose levels were decreased in K-KO-K mice compared with K-LL-K mice; when the offspring of a KO mouse were fed by a LL mother (K-LL-L and K-KO-L), blood glucose levels were no longer reduced (Fig. 7E) (Fig. 7E: *n* = 4–7, one-way ANOVA; F (7, 35) = 1.433, *p* = 0.2238) (*post hoc* comparison, Tukey’s multiple comparisons test). Furthermore, the blood glucose level in the offspring of a LL mother did not significantly differ among the different mouse groups (Fig. 7E). Consistent with these findings, the GTT and ITT showed that feeding from a KO mother significantly increased the insulin sensitivity of L-LL and L-KO offspring when challenged with a HF diet (Fig. 7F and J) (Fig. 7J: *n* = 4–6, one-way ANOVA; F (7, 33) = 3.369, *p* = 0.008) (*post hoc* comparison, Tukey’s multiple comparisons test, *p < 0.05), but glucose tolerance was not affected (Fig. 7G and K). The insulin sensitivity of K-LL and K-KO offspring was not affected by the genotype of their foster mothers (Fig. 7H and J). However, when the foster mother was a LL mouse, the offspring of the KO mice (K-LL-L and K-KO-L) no longer exhibited any increases in glucose tolerance as K-KO-K mice (Fig. 7I and K) (Fig. 7K: *n* = 4–6, one-way ANOVA; F (7, 35) = 2.503, *p* = 0.034) (*post hoc* comparison, Tukey’s multiple comparisons test, *p < 0.05).

## Discussion

The anti-obesity function of GH has broad implications for the maintenance of metabolic equilibrium. Evidence from the *Ghr* deficient mouse model and clinical studies revealed the critical role of GH signaling in lipid and glucose homeostasis [15, 20, 22, 36]. However, the role of GH signaling in intergenerational adipose development is poorly understood. In particular, the sexual dimorphism of the adipose GH axis on fat deposition and lipid metabolism has not been fully elucidated. The present study provides new insights into the sex-specific effects of maternal adipose-specific *Ghr* deficiency on reproductive function and offspring metabolic health. Our results demonstrate that adipose-specific *Ghr* deficiency increases body fat mass and disrupts reproductive function in female KO mice. In addition, we identified potential sex-dependent differences in transgenerational programming affecting glucose and lipid metabolism, highlighting the complex role of GH in modulating sex differences in both reproductive health and metabolic homeostasis.

First, we found that the tissue-specific knockout of *Ghr* in adipose tissue significantly impaired reproductive function in female mice. This aligns with existing evidence that GH influences ovarian function. Although most global *Ghr* gene disrupted (GHRKO) mice are fertile, the age at first conception is greatly delayed. In pregnant GHRKO females, fetal size is reduced, and pregnancy is prolonged. The litter size and body weight of offspring are also significantly lower in GHRKO mice than in normal mice [37, 38]. Moreover, total *Ghr* knockout induces hyperprolactinemia and affects gonadal functions in male mice, likely through GH resistance and a reduction in peripheral IGF-1 levels [39]. In the present study, a lack of GH action in adipose tissues resulted in significant defects in the reproductive function of female KO mice. The pregnancy and fertility rates of female KO mice were both significantly lower than those of LL mice, while the reproductive capacity of male KO mice seemed to be unaffected. In humans, obese women suffer longer times to conception, lower fertility rates, and greater rates of miscarriage [40]. Specifically, obesity alters hormones such as adiponectin and leptin, affecting all levels of these hormones within the hypothalamic-pituitary-gonadal (HPG) axis [41, 42]. Imbalances in adipokine levels can affect ovarian steroidogenesis, resulting in a disrupted hormonal environment [43]. Notably, our previous research showed that adiponectin levels did not differ between 20-week-old male KO and LL mice [22]. However, lower adiponectin levels in female KO mice, as observed in our study, may directly contribute to impaired folliculogenesis and reduced fertility, as adiponectin has been linked to ovarian function and oocyte quality [44]. In addition, the reduction in serum E2 and FSH levels in KO females indicates that adipose tissue, via hormonal and adipokine signaling (e.g., adiponectin), plays a crucial role in maintaining reproductive health. Our findings point to disrupted *Ghr* in adipose tissue as a key factor in altering the reproductive axis. The adipose GH-GHR axis may interact with the HPG axis in a sex-specific manner, leading to divergent reproductive phenotypes in males and females.

Second, our study demonstrated the impact of adipose-specific knockout of *Ghr* on the intergenerational development of adipose tissue. GH is well recognized for its role in the regulation of adipose tissue homeostasis. In normal physiological conditions, GH promotes the mobilization of lipids from adipose stores by stimulating lipolysis, reducing fat accumulation [26]. By knocking out *Ghr* in adipose tissue, we interrupted this signaling pathway, leading to notable alterations in adipocyte size and fat mass. Importantly, the effect of this disruption was not confined to the maternal generation but also impacted the fat development of the offspring. We found that *Ghr* deficiency in obese mothers blocks the genetic predisposition to obesity in female, but not male offspring. The differential impact of maternal *Ghr* deficiency on male and female offspring underscores the importance of sex-specific developmental programming. Similarly, a recent clinical study showed that the association between the fat mass of mothers and offspring is stronger than that between fathers and offspring; this was especially true for the association between mothers and daughters but not between mothers and sons [45]. In rodents, the growth and adipogenesis of adipose tissue primarily occur in the last week of gestation and accelerate during early postnatal life until pups are completely weaned [46]. The reversal of the protective metabolic effects in female offspring when fostered by control mothers suggests that early postnatal factors, such as breast milk composition and maternal care, play a critical role in shaping the offspring’s metabolic phenotype [47]. Adipokines such as leptin and adiponectin are critical for regulating energy balance and adipogenesis. Although our study did not directly measure these adipokines in the breast milk, it is well established that maternal adipokines can be transferred to the offspring through breastfeeding, impacting their metabolic outcomes [48, 49]. Furthermore, the observed sexual dimorphism in response to maternal *Ghr* deficiency may also be mediated by differential epigenetic programming in adipose tissue [50]. Female offspring of KO mothers exhibited persistent upregulation of thermogenic genes in BAT, which may be driven by sex-specific DNA methylation or histone modifications established during early development [51, 52]. Our data demonstrated that GH in adipose tissue plays a key role in intergenerational effects on adipose development, but future studies are warranted to confirm the underlying mechanisms involved.

Finally, our study demonstrated a beneficial effect of adipose-specific *Ghr* knockout on lipid and glucose metabolism in female KO mice and their offspring. It has been shown that *Ghr* global null mice exhibit dwarfism, severe postnatal growth retardation and obesity, but with greatly enhanced insulin sensitivity [20]. Similarly, our previous research has shown that removal of GH action in adipose tissue also improves insulin sensitivity and reduces insulin levels in male KO mice under high-fat die, though the serum blood glucose levels do not display any notable differences between 20-week-old male KO and LL mice under regular chow [22]. Here we found that fasting blood glucose levels in female KO mice were significantly lower than in female LL mice. Furthermore, female offspring of KO mothers exhibited improved glucose metabolism, reduced hepatic steatosis, and resistance to diet-induced obesity, in contrast to their male counterparts, who showed no similar metabolic benefits. This highlighting a clear sexual dimorphism in the metabolic response to GH signaling. This sexual dimorphism in metabolic outcomes may be driven by differences in adipose tissue distribution, thermogenic capacity, and hormonal regulation. The distribution of adipose tissue differs between sexes, with females having a higher proportion of subcutaneous adipose tissue, which is more metabolically active and insulin-sensitive than visceral fat [53]. This could partly explain the enhanced metabolic benefits observed in female offspring. Additionally, studies have demonstrated that women generally have higher mass and activity of BAT compared to men [54, 55]. The upregulation of thermogenic genes such as *Ucp1* and *Pgc1α* in the BAT of female offspring suggests enhanced BAT activity and energy expenditure, which are known to protect against obesity and glucose intolerance [51]. The sexual dimorphism in KO mice could also be driven by differences in sex hormones, which modulate lipid metabolism [56]. Adipose tissue is a major source of estrogens and increases in proportion to total body adiposity [57]. Adipocyte express estrogen receptors, and GH has well-established interactions with these sex steroids [58, 59]. The absence of *Ghr* in adipose tissue may shift the metabolic balance toward greater energy expenditure through enhanced thermogenesis at lower hormone levels [56]. In contrast, testosterone in male offspring may blunt the effects of *Ghr* disruption on thermogenesis, leading to the absence of metabolic benefits in males. Furthermore, maternal *Ghr* deficiency may induce epigenetic modifications in genes involved in glucose metabolism and lipid storage, creating a lasting metabolic advantage in female offspring that is not present in males [60]. Beyond adipose tissue, the loss of growth hormone signaling in the liver and islets has also shown notable sex differences in gene expression and metabolic processes [61–63]. The sex-specific nature of these effects underscores the complex role of GH in metabolic regulation and highlights the potential for targeting GH pathways to treat metabolic disorders in a sex-specific manner.

### Perspectives and ssignificance

This study provides novel insights into the role of GH signaling in adipose tissue and its far-reaching impact on both reproductive health and metabolic regulation, including intergenerational effects. By demonstrating sex-specific metabolic outcomes in the offspring of *Ghr*-deficient mothers, this work highlights the complexity of hormone signaling pathways in developmental programming and adipocyte biology. The results suggest that targeting adipose *Ghr* may offer new therapeutic strategies for mitigating metabolic diseases, particularly in populations with obesity-related reproductive and metabolic dysfunctions. In addition, the identification of sexually dimorphic responses points toward the need for personalized medicine approaches that consider sex differences in treatment efficacy. Future research should explore the underlying molecular mechanisms of these effects and evaluate whether similar protective outcomes can be replicated in human models to offer translational potential.

## Limitatsions

There are some limitations to this study. Although the study touches on the role of adipokines, it does not include direct measurements of key adipokines (e.g., leptin, adiponectin) in breast milk or circulation, which could provide additional insights into how maternal adipose *Ghr* deficiency impacts offspring development through hormonal signaling. Additionally, given recent evidence on the role of gut microbiota in metabolic health and intergenerational inheritance, it will be interesting to investigate microbiome changes in the offspring of KO and LL mice. This would help clarify whether gut microbiota play a role in mediating the observed metabolic differences. Future research addressing these areas could provide a more comprehensive understanding of the intergenerational effects of GH signaling in adipose tissue on metabolic health.

## Conclusion

In conclusion, our results demonstrate that disruption of GH signaling in adipose tissue leads to distinct phenotypic outcomes in male and female mice, highlighting the complex role of GH in modulating sex differences. The sex-specific impact of *Ghr* deficiency in this study was most pronounced in reproductive and metabolic health, where female mice showed a unique susceptibility to reproductive dysfunction but also greater resilience against diet-induced obesity and glucose intolerance. In contrast, male mice displayed minimal changes in reproductive function and did not benefit metabolically from maternal *Ghr* deficiency. These findings suggest that GH acts as a key regulator of sex-specific physiological pathways, potentially through differential modulation of sex hormones, adipokine profiles, and thermogenic capacity. Understanding the molecular and epigenetic mechanisms underlying these sex-specific responses will be crucial for developing targeted interventions for maternal obesity and associated metabolic disorders. Future studies are necessary to further elucidate the mechanisms underlying these sexually dimorphic phenotypes, including the roles of sex hormones and adipose-derived signals in modulating metabolic outcomes. Additionally, future research should explore the impact of early postnatal factors, such as breast milk composition and gut microbiota, in mediating these intergenerational effects to develop effective strategies for optimizing offspring health.

## Electronic supplementary material

Below is the link to the electronic supplementary material.

Supplementary Material 1

## Electronic supplementary material

Below is the link to the electronic supplementary material.

Supplementary Material 1

## Data Availability

No datasets were generated or analysed during the current study.
