# Supplementary material for: Sexual dimorphism in the effects of maternal adipose tissue growth hormone receptor deficiency on offspring metabolic health
Source: Biol Sex Differ. 2024 Dec 2;15:98. doi: 10.1186/s13293-024-00676-2 (PMC11610217; doi:10.1186/s13293-024-00676-2)
Supplement: Supplementary file 1 — Supplementary Material 1 [file 13293_2024_676_MOESM1_ESM.docx]

**Supplemental Information**

**Table S1 Detailed description of the mouse line**

| **Common Name** | **Description** | **Name in this paper** |
| --- | --- | --- |
| *Tg(Adipoq-cre)1Evdr*  (JAX stock #010803) | Mice harboring the Adipoq-Cre BAC transgene express Cre recombinase under control of the mouse adiponectin (Adipoq) promoter/enhancer regions within the BAC transgene. | Adipoq-Cre |
| *Ghr^flox/flox^* | Mice possess LoxP sites flanking exon 4 of the growth hormone receptor (*Ghr*) gene [1] | LL |
| *Ghr^wt/flox^Tg(Adipoq-cre)1Evdr* | Heterozygote offspring of *Ghr^flox/flox^* and Adipoq-cre | *Ghr^WL^*(+) |
| *Ghr^flox/flox^Tg(Adipoq-cre)1Evdr* | Mice with deletion of Exon4 sequence in *Ghr* gene of adipose tissue | KO |

**Table S2 Abbreviation description of the offspring mice**

| **Abbreviation** | **Detailed Description** |
| --- | --- |
| L | LL |
| K | KO |
| L-LL | LL offspring of LL maternal parent |
| K-LL | LL offspring of KO maternal parent |
| L-KO | KO offspring of LL maternal parent |
| K-KO | KO offspring of KO maternal parent |
| L-LL-L | LL offspring of LL maternal parent fed by LL mother |
| L-KO-L | KO offspring of LL maternal parent fed by LL mother |
| K-LL-L | LL offspring of KO maternal parent fed by LL mother |
| K-KO-L | KO offspring of KO maternal parent fed by LL mother |
| L-LL-K | LL offspring of LL maternal parent fed by KO mother |
| L-KO-K | KO offspring of LL maternal parent fed by KO mother |
| K-LL-K | LL offspring of KO maternal parent fed by KO mother |
| K-KO-K | KO offspring of KO maternal parent fed by KO mother |

**Table S3 The composition of research diets**

|  | **XTCON50J** | | | **XTHF60** | | |
| --- | --- | --- | --- | --- | --- | --- |
|  | gm% | Kcal/kg | Kcal% | gm% | Kcal/kg | Kcal% |
| Proteins | 18.8 | 752 | 18.14 | 23.25 | 930 | 20.54 |
| Fat | 5.2 | 468 | 60.65 | 34.55 | 3110 | 12.79 |
| Carbohydrates | 61.0 | 2440 | 21.22 | 27.02 | 1088 | 66.67 |
| Total |  | 5128 | 100 |  | 3660 | 100 |

**Reference**

[1]. Wu, Y., C. Liu, H. Sun, A. Vijayakumar, P.R. Giglou, R. Qiao, et al., Growth hormone receptor regulates β cell hyperplasia and glucose-stimulated insulin secretion in obese mice [J]. J Clin Invest, 2011. 121(6): p. 2422-6.
