# Supplementary material for: Sexual dimorphism in the effects of maternal adipose tissue growth hormone receptor deficiency on offspring metabolic health
Source: Biol Sex Differ. 2024 Dec 2;15:98. doi: 10.1186/s13293-024-00676-2 (PMC11610217; doi:10.1186/s13293-024-00676-2)
Supplement: Supplementary file 2 — Supplementary Material 1 [file 13293_2024_676_MOESM2_ESM.docx]

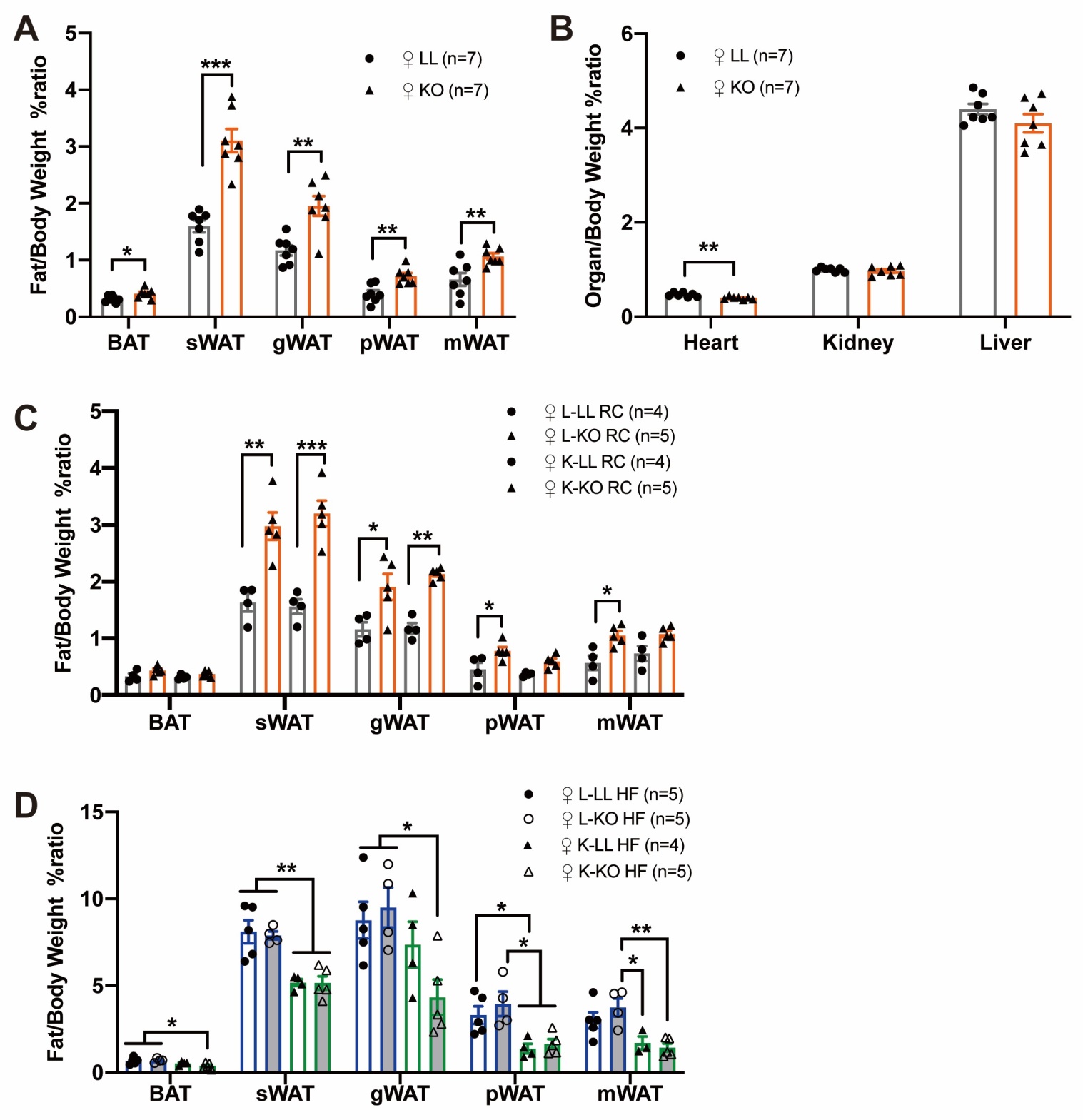


**Figure S1: The ratio of fat depots and other organs to body weight**

**A:** Fat tissue indices of 24-week-old female KO and LL mice (n = 7 mice/group). Related to Fig. 1C.

**B:** Organ indices of 24-week-old female KO and LL mice (n = 7 mice/group). Related to Fig. 1D.

**C:** Fat tissue indices of female offspring of KO and LL maternal mice under RC feeding (n = 4–5 mice/group). Related to Fig. 3C.

**D:** Fat tissue indices of female offspring of KO and LL maternal mice after high-fat diet feeding for 16 weeks (n = 4–5 mice/group). Related to Fig. 5A.

All the values are presented as the means ± SEMs. The statistical significance was indicated by **p* < 0.05, ***p* < 0.01 and ****p* < 0.001.


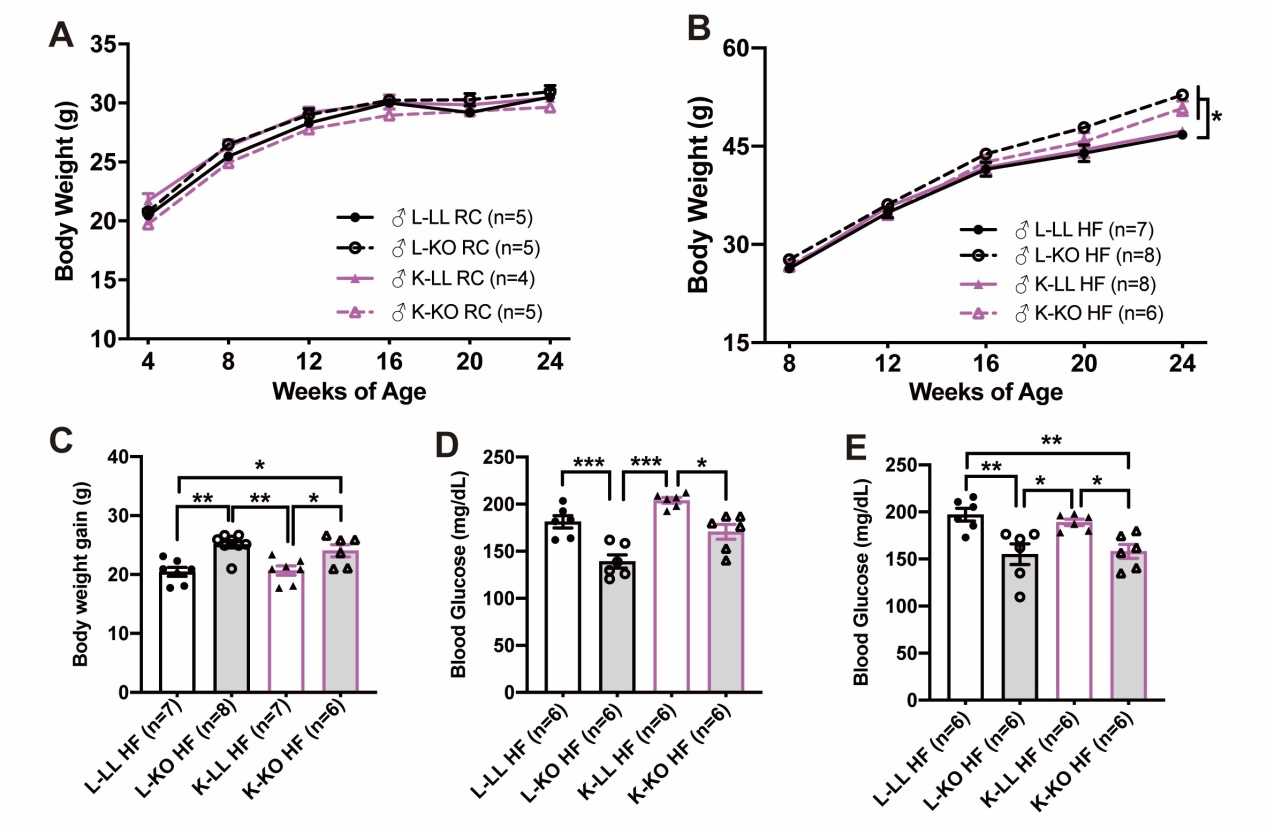


**Figure S2: Genotype of maternal mice did not affect body weight and glucose homeostasis in male offspring**

**A:** Growth curves of male offspring of KO and LL maternal mice during RC feeding (n = 4–5 mice/group).

**B:** Growth curves of male offspring of KO and LL maternal mice during HF feeding (n = 6–8 mice/group).

**C:** Increase in body weight (24-week *vs.* 8-week) of HF feeding male offspring of KO and LL maternal mice (n = 6–8 mice/group).

**D:** Fasting blood glucose levels of male offspring mice fed a high-fat diet for 8 weeks (n = 6–8 mice/group).

**E:** Fasting blood glucose levels of male offspring mice fed a high-fat diet for 16 weeks (n = 6–8 mice/group).

All the values are presented as the means ± SEMs. The statistical significance was indicated by **p* < 0.05, ***p* < 0.01 and ****p* < 0.001.
